# In Vitro Studies on Nasal Formulations of Nanostructured Lipid Carriers (NLC) and Solid Lipid Nanoparticles (SLN)

**DOI:** 10.3390/ph14080711

**Published:** 2021-07-23

**Authors:** Cláudia Pina Costa, Sandra Barreiro, João Nuno Moreira, Renata Silva, Hugo Almeida, José Manuel Sousa Lobo, Ana Catarina Silva

**Affiliations:** 1 UCIBIO/REQUIMTE, MEDTECH, Laboratory of Pharmaceutical Technology, Department of Drug Sciences, Faculty of Pharmacy, University of Porto, 4050-313 Porto, Portugal; claudiaspinacosta@gmail.com (C.P.C.); hperas5@hotmail.com (H.A.); slobo@ff.up.pt (J.M.S.L.); 2 UCIBIO/REQUIMTE, Laboratory of Toxicology, Department of Biological Sciences, Faculty of Pharmacy, University of Porto, 4050-313 Porto, Portugal; sandrafcbarreiro@gmail.com (S.B.); rsilva@ff.up.pt (R.S.); 3 CNC—Center for Neuroscience and Cell Biology, Center for Innovative Biomedicine and Biotechnology (CIBB), Faculty of Medicine (Pólo I), University of Coimbra, 3004-504 Coimbra, Portugal; jmoreira@ff.uc.pt; 4 UC—University of Coimbra, CIBB, Faculty of Pharmacy, Pólo das Ciências da Saúde, Azinhaga de Santa Comba, 3000-548 Coimbra, Portugal; 5 FP-ENAS (UFP Energy, Environment and Health Research Unit), CEBIMED (Biomedical Research Centre), Faculty of Health Sciences, University Fernando Pessoa, 4249-004 Porto, Portugal

**Keywords:** nasal administration, nanostructured lipid carriers, solid lipid nanoparticles, in vitro cell cultures, 3D nasal casts

## Abstract

The nasal route has been used for many years for the local treatment of nasal diseases. More recently, this route has been gaining momentum, due to the possibility of targeting the central nervous system (CNS) from the nasal cavity, avoiding the blood−brain barrier (BBB). In this area, the use of lipid nanoparticles, such as nanostructured lipid carriers (NLC) and solid lipid nanoparticles (SLN), in nasal formulations has shown promising outcomes on a wide array of indications such as brain diseases, including epilepsy, multiple sclerosis, Alzheimer’s disease, Parkinson’s disease and gliomas. Herein, the state of the art of the most recent literature available on in vitro studies with nasal formulations of lipid nanoparticles is discussed. Specific in vitro cell culture models are needed to assess the cytotoxicity of nasal formulations and to explore the underlying mechanism(s) of drug transport and absorption across the nasal mucosa. In addition, different studies with 3D nasal casts are reported, showing their ability to predict the drug deposition in the nasal cavity and evaluating the factors that interfere in this process, such as nasal cavity area, type of administration device and angle of application, inspiratory flow, presence of mucoadhesive agents, among others. Notwithstanding, they do not preclude the use of confirmatory in vivo studies, a significant impact on the 3R (replacement, reduction and refinement) principle within the scope of animal experiments is expected. The use of 3D nasal casts to test nasal formulations of lipid nanoparticles is still totally unexplored, to the authors best knowledge, thus constituting a wide open field of research.

## 1. Introduction

The nasal route had been widely used for several years for the local treatment of nasal diseases, through the administration of corticosteroids, decongestants, and antihistamines [1]. More recently, the possibility of reaching the brain through the nose without the need to cross the blood−brain barrier (BBB) has gained attention, especially to improve the treatment of central nervous system (CNS) disorders, including epilepsy, Alzheimer’s disease, Parkinson’s disease, multiple sclerosis, gliomas, among others [2,3]. As the BBB is vital to protect the brain from exogenous substances, it acts as an obstacle to the passage of most drugs. This barrier is a semipermeable membrane that maintains the CNS homeostasis, providing nutrient exchange between the brain and the blood. The presence of tight junctions of endothelial capillary cells restricts the passage of drugs, with smaller molecules weighing less than 400 Da and lipophilic molecules being the only ones that can easily cross this barrier [4,5,6,7,8].

Different strategies have been investigated to increase the drug passage through the BBB, such as electromagnetic force-field techniques and mini-pump-assisted intracranial delivery. However, these methods are invasive and can lead to the passage of toxins to the brain, being nonselective and neurotoxic [9,10,11]. Thus, it is essential to find new ways to avoid the need to bypass the BBB to target drugs to the brain. In this area, intranasal administration has emerged as the only direct drug delivery route to the brain via the olfactory and trigeminal nerves, without the need to pass into the systemic circulation and cross the BBB. Nonetheless, it is important to keep in mind that after intranasal administration, part of the drug is absorbed into the systemic circulation and reaches the brain through the BBB [3]. In addition, the use of lipid nanoparticles, such as solid lipid nanoparticles (SLN) and nanostructured lipid carriers (NLC), to promote the targeting of drugs to the brain after intranasal administration, has been suggested as a promising alternative to the conventional treatments of brain disorders [1,3,12,13]. 

This review provides the state of the art of the in vitro studies with nasal formulations containing lipid nanoparticles, reported in the past two years. The manuscript starts with anatomical and physiological considerations of the nasal route, followed by the requisites of nasal formulations. Subsequently, the most used in vitro cell models for performing studies with nasal formulations, and relevant outcomes observed with liquid and semisolid nasal formulations of SLN and NLC are described. Finally, the use of in vitro nasal cavity and computational models to predict the in vivo performance of nasal formulations is reported.

## 2. Nasal Route

### 2.1. Anatomical and Physiological Considerations

**Figure 1:**
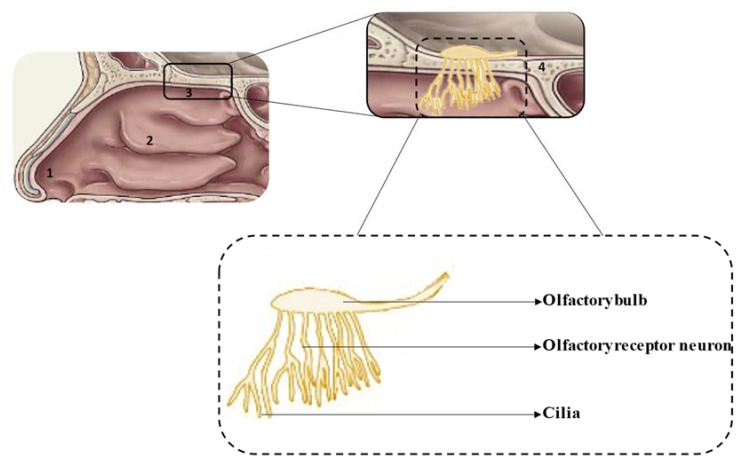
Schematic representation of the nasal cavity (**top**) and olfactory region (**bottom**): 1—vestibule, 2—respiratory region, 3—olfactory region, 4—cribriform plate.

The anatomical and physiological characteristics of the different regions of the nasal cavity are summarized in Table 1 and the location of each region is shown in Figure 1 [12,13,14]. 

### 2.2. Nose-to-Brain Delivery

**Figure 2:**
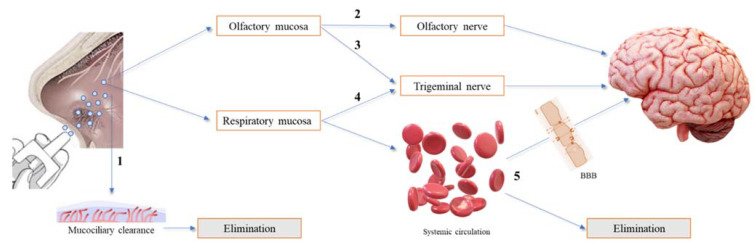
Overview of the different drug pathways after nasal administration. (1) The drug is eliminated by the mucociliary clearance mechanism. (2) The drug reaches the olfactory mucosa, passes through the olfactory nerve, via intraneuronal and/or extraneuronal transport, and reaches the brain. (3) The drug reaches the olfactory mucosa, passes through the trigeminal nerve and reaches the brain via the cribriform plate. (4) The drug reaches the respiratory mucosa, passes through the trigeminal nerve and reaches the brainstem. (5) The drug reaches the respiratory mucosa, is absorbed into the systemic circulation, and diverges between passage to the brain, upon crossing the blood−brain barrier (BBB), and elimination, before reaching the brain.

After nasal administration, different pathways of drug transport from the nose to the brain can occur, which have been divided into direct transport, indirect transport and a combination of both. Besides, some drug can be eliminated by the mucociliary clearance mechanism before reaching the olfactory or/and respiratory regions. To our knowledge, there is no confirmation of the exact transport mechanism followed by intranasal drugs, which seems to be influenced by the drug’s molecular characteristics, formulation consistency (liquid or semi-solid) and type of application device. Thus, it is impossible to assess the exact amount of drug reaching the brain after intranasal administration via a specific transport mechanism, although good approaches have been reported in in vivo studies that compared the results of the amount of drug reaching the brain after intranasal and intravenous administrations. In addition, toxicological concerns were raised related to the possibility of an accumulation of excipients in the brain and the risk of impairment of the mucociliary clearance mechanism. Figure 2 summarizes the different drug pathways after nasal administration [2,3,6,18].

### 2.3. Requisites of Nasal Formulations

Some factors of the nasal formulations can interfere with drug absorption and should be considered. For instance, these formulations should be isotonic (i.e., osmolality between 280 mOsm/Kg and 310 mOsm/Kg) and have a pH close to that of the nasal cavity (5.0–6.8), to avoid discomfort, mucosal irritation and/or damage to the cilia, after administration [13,19]. In addition, the drug excipients used should be compatible with the nasal mucosa to avoid irritation and toxicity [20,21].

One of the main disadvantages of intranasal administration is the rapid elimination of the drug through mucociliary clearance (a physiological defense mechanism that eliminates foreign substances every 15–30 min). To avoid this, substances that interact with the mucus can be added to the formulations. The mucus is composed of water, mucin and other proteins, electrolytes, enzymes and lipids [15]. Mucin is a negatively charged glycoprotein and, therefore, positively charged formulations can easily bind it through electrostatic interactions, which facilitates mucoadhesion. In contrast, negatively charged formulations can penetrate mucin chains and hydrogen bonds can be formed, which improves mucoadhesion [13,22].

New strategies to overcome the drawbacks of the nasal formulations have been investigated. For example, the use of nanocarriers, such as lipid nanoparticles, to achieve prolonged release, protection against enzymatic degradation and improve targeting to the brain [23]. The use of permeation enhancers, including mucoadhesive polymers and in situ hydrogels to improve drug retention time in the nasal mucosa and, consequently, drug absorption is also a commonly used strategy [23]. There are already marketed nasal formulations (e.g., Nasonex and Rhinocort) that increase viscosity after administration, improving the retention time of the drug in the nasal cavity [13].

## 3. In Vitro Cell Models to Evaluate the Effectiveness of Nasal Formulations

There are different models available to assess the nose-to-brain drug delivery that can be used to determine the drug absorption and permeability through the nasal cavity, to evaluate its pharmacokinetic, toxicity and possible drug transport interactions [24]. Indeed, different in vitro, in vivo and ex vivo models are widely used to assess nose to brain drug transport [24]. While in vivo models allow nasal absorption and pharmacokinetic studies and ex vivo models allow the performance of nasal perfusion studies, in vitro models are useful to predict drug permeability, allowing exploration of the underlying mechanism(s) of drug absorption and transport through the nasal route [24,25]. 

Regardless of the remarkable information that can be achieved from in vivo studies, the extrapolation of the results to humans is still challenging given the significant differences between species in what concerns the structure of the upper airway and the epithelial cell populations of the mucosal surface tissue covering the nasal routes. Additionally, in vivo studies often require a large number of animals and higher drug amounts [26].

Recently, many distinct nasal in vitro models have been developed. Accordingly, cell culture models and excised nasal mucosae are important and useful in in vitro models to study the characteristics underlying the metabolic barrier capacity of the nasal epithelium, and for drug permeability studies [27,28]. Moreover, by using cultured human nasal epithelial cells (both primary cells and/or immortalized cell lines) an accurate prediction of the drug metabolism, toxicity and transport across the nasal tissue in humans may be successfully accomplished and may even provide results with a more direct clinical relevance [26,29]. In fact, the use of standardized in vitro nasal epithelial cell cultures in pharmacological and toxicological studies offers diverse advantages, including the possibility of controlling/monitoring experimental conditions, the potential exclusion of pre- and post-mucosal issues, the execution of a quicker and more efficient evaluation of permeability, metabolism and toxicity and their underlying mechanisms, and the limited needs of animal studies, therefore reducing costs [26,27]. However, in vitro models for nasal drug delivery studies remain often imperfect, as they still lack a cell line that adequately mimics the nasal epithelium [29,30].

In vitro cell culture models are an ideal alternative for permeability screening studies. They present several advantages when compared with other models, namely in situ or in vivo models, as they allow the rapid evaluation of the permeability profiles of a given drug, and the possibility to test molecules that could be harmful if tested directly in vivo [25]. Additionally, the use of in vitro models with human cells does not involve the same ethical problems and regulatory impediments as the studies performed with in vivo models [25,30].

The efficacy of a drug administered in the nasal cavity will depend on the anatomic region where its absorption takes place, between the nasal epithelium and the lungs [26]. Nasal cells or tissue excised from the nasal cavity may be originated from different nasal domains, including the vestibular area, the atrium, the turbinates (superior, medium and inferior) and the olfactory epithelium [27]. To perform transport and metabolic studies, the respiratory epithelium, a pseudostratified ciliated columnar epithelium in the region of the medium and inferior turbinates, is the most relevant [27]. This region has a particular architecture that highly influences the absorption of a given drug, as it holds ciliated columnar cells with many mitochondria in the apical side, basal cells, nonciliated columnar cells with microvilli, goblet cells with mucous granules and a fully developed Golgi apparatus [27]. 

At the nasal mucosa, the absorption of intranasally administered drugs is highly influenced by both passive diffusion and carrier-mediated transport processes. The transporters here implicated belong to the two most important families of transporter proteins: the ATP binding cassette (ABC) and the solute carrier (SLC) superfamilies. ABC transporters, efflux pumps including P-glycoprotein (P-gp, encoded by the *ABCB1* gene) and multidrug resistance-associated proteins (MRPs), represent a family of transporters that uses the energy resulting from ATP hydrolysis to carry their substrates across biological membranes and against their concentration gradients [31,32]. Additionally, the SLC gene family translates a wide group of protein membrane carriers that are present in many organelle or cellular membranes. This transporter superfamily includes several passive transporters, ion-coupled transporters and exchangers. Nevertheless, the information available on the expression and functionality of the SLC transporters in the human nasal mucosa, as well as in the existing nasal in vitro models, remains fairly scarce when compared with data reported in other epithelial barriers and tissues, such as in the liver, intestine or lungs [31].

In order to study the permeability of drugs through the respiratory epithelium, and its underlying mechanisms, several in vitro models are used, including excised tissue (human, bovine, porcine, and others), primary human nasal epithelial cell cultures and immortalized cell lines [28,30,33,34]. Excised tissues represent the closest physiological approach in terms of histology, transporter expression and cell type distribution [35,36]. In fact, both excised tissue (organotypic explants) and primary cell cultures are morphologically closer to the airway mucosa, presenting proper tissue architecture and differentiation, but the poor accessibility, the lack of standardization and the existing interspecies differences (nonhuman alternatives) limits their applicability in drug transport and permeability studies [30,33,34,37,38]. In addition, the reproducibility of organotypic explants and primary cell cultures is often challenging due to the genetic variability, inter- and intraindividual differences between the donors, and the uncontrollable environmental variables preceding tissue harvest. They have complex isolation procedures and limited lifespan, are difficult to maintain in culture, time-consuming, less reproducible, and more expensive [28,30,33]. On the other hand, immortalized cell lines are easily maintained in culture and offer higher reproducibility and genetic homogeneity, being the most convenient in vitro models of the nasal epithelial barrier, even though lacking the real organ complexity originated by the presence of cilia, mucus and blood vessels [35]. However, although epithelial cell lines are the most used for drug transport studies, there still is a lack of cell lines that completely mimic the nasal epithelium [28,30,33]. Currently, the available immortalized nasal cell lines include the NAS 2BL, a rat nasal epithelial tumor cell line, BT cell line, bovine turbinate obtained from new-born bovine turbinate tissue, and RPMI 2650 cells, this last one being the only human nasal cell line that could be properly used for drug transport studies [26,35,39]. Because of this deficiency, and despite the known differences in the morphologies of different cell lines, many research groups have been using bronchial epithelial cells as a surrogate for nasal epithelial cells, such as the human bronchial epithelial cell line Calu-3 [26,29].

The choice of a proper cell culture model is influenced by different factors, including the level of cell differentiation required. In addition, the selected cell culture techniques (air−liquid interface or immersion cultures) and cell growth conditions (seeding density, cell confluency, media supplements, culture periods, cell culture on a collagen coating) certainly may influence the cells’ phenotype differentiation, and their morphological and functional features. As a result, the expression of drug transporters or drug metabolizing enzymes may be different from the human respiratory epithelium, altering the permeability profile of a drug [25,27,40]. 

As mentioned, the cell culture conditions will highly influence the results of permeability and transport, metabolism, or toxicity studies. For instance, the cell-support membranes (uncoated or coated extracellular matrix) selected for permeability studies, the cells’ electrical properties, the cellular confluency and tight junction generation, the differentiation pattern of the cell monolayer (morphologically well differentiated with ciliated, nonciliated and secretory cells), and the developed ciliary activity, mucus secretion, and metabolic activity, [26,40]. The nasal epithelium morphologically and functionally resembles the respiratory epithelium of the lower airways, which can be useful to culture differentiated nasal, tracheal or bronchial epithelium cells [26].

The cell culture models can be compared, and their integrity assessed through the evaluation of the permeation coefficients of different marker compounds and the transepithelial electrical resistance (TEER) determination, which indicates tight junction development [26,28]. For instance, the human nasal mucosa obtained from the inferior turbinates demonstrated TEER values of ranging from 40 up to 120 Ω cm^2^, thus being moderately different from excised animal tissues (from 90 up to 180 Ω cm^2^) [30,34]. In addition, and when compared with human nasal excised tissues, primary cell lines often yield more tight junctions with significantly higher TEER values (600–3100 Ω cm^2^) [34,36]. This difference in the TEER values could lead to an underestimation of the permeability, particularly of more hydrophilic compounds, typically transported by paracellular pathways [26].

The common pathways for drug transport across the nasal epithelium are similar to other epithelia in the body, being the two main routes involved in transepithelial drug permeability across the nasal epithelium, the transcellular and the paracellular transport pathways [26]. Thereby, in vitro cell culture models are a useful tool to discriminate passive and active transepithelial drug transport [26].

### 3.1. Human Nasal Epithelial Cells (HNEpC)

Human nasal epithelial cells (HNEpC) can be obtained from the nasal tissues of patients submitted to endonasal surgery of polyps, septum deviation, hyperplastic conchae or even nasal reconstruction [26]. Primary cell cultures of HNEpC present some disadvantages related to the shortage of human nasal tissue available from one donor, the short-term cultures, the heterogeneity within cultures and between cell cultures, significant variability between donors and cell culture difficulties [26,31,37,40]. Nevertheless, and although respiratory epithelial cells can only be passed two or three successive times, these cells can be subcultured into confluent monolayers, while retaining the capacity to differentiate into ciliated and secretory cells [26,37,40].

Primary cell cultures are the most reliable in resembling the native airway epithelium and, therefore, are suitable for drug transport studies [40]. HNEpC retain morphological and functional characteristics similar to the native human nasal epithelium, showing mucin secretion, expression of mucins (MUC5AC, MUC5B and MUC8), microvilli and cilia, aminopeptidase, and tight junction proteins [40]. However, the use of different media, culture interfaces, and time in culture have substantial consequences on the human nasal cell ultrastructure, barrier formation, and transporter expression [40]. For example, the use of liquid cell cultures of primary HNEpC allows the formation of monolayers of simple cuboidal cells, while when cultured at an air−liquid interface, the same HNEpC cells can differentiate into multilayers similar to the original nasal tissues as far as structure, mRNA and immune responses are concerned [37].

### 3.2. Human Nasal Septum Quasidiploid Tumour Cells (RPMI 2650)

The human nasal septum quasidiploid tumor cells (RPMI 2650) were initially obtained, in 1962, from an anaplastic squamous cell carcinoma of the human nasal septum [26,35,41]. RPMI 2650 cells are often used to study drug metabolism and toxicity as they produce different cytokeratins, retain the ability for mucus secretion and exhibit identical metabolic activity to the human nasal mucosa. Furthermore, these cells are quite stable throughout continued passaging, maintaining their quasidiploid karyotype in culture [24,27,29,35]. 

Depending on the selected cell support or extracellular matrix, RPMI 2650 cells in culture may form clusters of round and slightly flattened cells, or may tend to spread [28]. When compared to excised human nasal tissue, these cells present similar aminopeptidase activity, expressing lysosomal aminopeptidase, leucine aminopeptidase and aminopeptidases N, A and B, enzymes that often influence the nasal permeability of peptides and proteins [26].

The RPMI 2650 cells were initially shown to be poorly differentiated into goblet or ciliated cells, did not express tight junctions and lacked the cell polarization that is essential for nasal drug transport studies [28,29]. However, over the last years, the RPMI 2650 cell model has been also used for drug permeability studies, through the application of specific air–liquid interface and liquid-covered culture conditions that lead to the formation of a tight barrier and confluent monolayers [12,24,28,30,35]. The apical and basolateral sides of the liquid-covered cell culture model are filled with culture medium, showing the presence of flattened ciliated cells and mucin expression [12,24]. In addition, in the air–liquid interface cell model, initially the apical and basolateral sides are filled with culture medium, and the apical side is aerated later. Moreover, the culture medium of the basolateral side is changed on alternate days, which creates a high similarity with living nasal tissue. This model contains ciliated cells, a high expression of mucin genes, expresses tight junction proteins and develops sufficient transepithelial electrical resistance, providing an adequate environment for cytotoxicity and permeability screening studies [12,24,28,30]. 

Overall, the RPMI 2650 cell model has similar physiologic barrier properties particularly for passive transport, and it has been the only human cell line used for nasal drug transport studies [28,30]. Upon specific culture conditions, the RPMI 2650 cell model has previously shown the ability of forming a permeable organotypic barrier with a tight uniform cell multilayer, exhibiting TEER values similar to the physiological, and permeation coefficients in the same range of those found in the human nasal mucosa [12,30].

The RPMI 2650 cell line expresses a variety of cell junction proteins, including ZO-1, occludin, claudin-1, E-cadherin, and β-catenin [36]. It was shown to moderately express genes encoding the multidrug resistant proteins (ABCB), being the most abundant ABCB6. Additionally, ABCC1 expression was the greatest amongst the multidrug resistance associated proteins (MRP/ABCC) [30]. In the human nasal respiratory mucosa, ABCC1 was present in ciliated epithelial cells with higher expression levels in serous glandular cells [30,31]. The RPMI 2650 cell line also expresses ABCB1 (P-gp), which is found in the normal mucosa of human nasal turbinates [30]. Concerning SLC transporters, the SLC19A2, SLC25A1, and SLC38A2 were the most abundant, and members of the SLC3 and SLC7 families, amino acid transporters, were found to be highly expressed (SLC3A1, SLC3A2, SLC7A6, SLC7A8, and SLC7A11), the SLC15A2 being well expressed in these cells [30].

Although more complex and difficult to handle and grow, co-cultures containing a collagen matrix of human nasal fibroblasts covered by a monolayer of RPMI 2650 cells have been developed to conduct transport and permeability studies. These co-cultures mimic the permeation barrier properties of nasal mucosa and simulate a non-pseudostratified and non-ciliated human nasal epithelium [28].

### 3.3. Human Lung Cancer Cells (Calu-3)

The human bronchial epithelial cell line Calu-3 represents a promising in vitro model of the upper airway epithelial barrier [36]. This submucosal adenocarcinoma cell line was obtained from the bronchial airways of a 25-year-old white Caucasian male. The Calu-3 cells are capable of forming differentiated, tight and polarized layers of a combined phenotype, including ciliated and secretory cells, have microvilli, express several cell junction proteins (tight junctions, desmosomes and zonulae adherens) and contain mucin granules [26,36]. Despite its origin, the Calu-3 cell line has characteristics similar to serous nasal cells, being useful for nasal permeability studies [42].

Regarding the culture conditions, studies have shown that the air−liquid culturing interface shows a closer resemblance of these cells to the in vivo airway epithelia, when compared to liquid−liquid culturing conditions, in terms of morphology, mucus production and barrier integrity, with TEER values close to the observed in primary human tracheo-bronchiolar cells [36]. At an air–liquid interface, Calu-3 cells form a confluent polarized cell monolayer with tight junctions and a uniform mucus layer [26].

The Calu-3 cell line provides an alternative in vitro model of the airway epithelia for drug permeability assessments, being easily maintained in culture, reproducible, with a wide passage range and ethical acceptability [36].

Calu-3 cell cultures may provide a valuable model for studying mucin gene expression and synthesis, electrolyte transport, epithelial barrier properties and their regulation mechanisms, as they highly express MUC1 and MUC5/5AC mRNA, and MUC5/5AC mucins, as well as functional cytochrome P450 isozymes (CYP1A1, CYP2B6 and CYP2E1) [26,42]. They can also be useful in permeability screening studies for the nasal and lung permeability potential of drugs [26,42].

### 3.4. Human Epithelial Colorectal Adenocarcinoma Cells (Caco-2)

The Caco-2 cell line was originally obtained from a human colon adenocarcinoma. Under normal culture conditions on semiporous filter membranes, these cells can differentiate into enterocytes. It is the most suitable model to study the absorption and permeability of drugs and drug formulations though the intestinal epithelium but is often used as a screening model to evaluate the nasal absorption of formulations after its differentiation [12,24,42].

Caco-2 cells can create polarized monolayers of columnar epithelial cells with brush border and tight junctions, improving TEER values. Among the advantages of these cells is the occurrence of both passive and active transports, including the expression of important uptake (SLC15A1, SLC22A1, SLC22A2, SLC22A3, SLCO2B1) and efflux transporters (P-gp/MDR1/ABCB1, BCRP/ABCG2, MRP2/ABCC2, MRP4/ABCC4) [43]. 

The Caco-2 cell model is widely used to assess the paracellular transport through the nasal epithelia. However, this model is unable to explain the effect of nasal mucus, mucins and clearance and physiological factors that interfere with drug permeability [24]. The same as other immortalized cell culture models, Caco-2 cells display heterogeneous populations that can lead to different permeabilities as a consequence of the cell source, number of cell passages, initial seeding density, transport experiment conditions, cell culture media, filter size and composition, and the transport buffer composition and pH. However, this variability can be reduced by standardization of the culture conditions and permeability assay [43].

### 3.5. Others

The Madin–Darby canine kidney (MDCK) cells, isolated from canine distal renal tissue (distal tubule epithelium), similarly to Caco-2 cells, differentiate into columnar epithelial cells and form tight junctions, when cultured on semiporous membranes. They have lower TEER and shorter culture times than Caco-2 cells [42,43]. MDCK cells express canine efflux transporters, namely Mdr1 (P-gp), Mrp1, Mrp2, Mrp5, and also functionally express uptake transporters, such as Oct2 (as expected in cells from renal origin), and transporters for monocarboxylic acids and peptides [43]. MDCK cells are a potential alternative to mimic the transport across the BBB because of the expression of P-gp and tight junction proteins, such as claudin-1, claudin-4 and occludin, which are important to form a restrictive paracellular barrier with tight junctions. Although useful, MDCK cells present several differences from the nasal mucosa, not being an ideal alternative for nasal permeability studies [42]. 

Concerning human bronchial epithelial cell lines, and similarly to the Calu-3 cell line, 16HBE14o- (16HBE) cell monolayers have been used as models of the airway epithelium due to their morphological characteristics, barrier properties and expression of drug transporters that are present in vivo [44,45]. The 16HBE cells are human bronchial epithelial cells firstly isolated from a 1-year-old male and then immortalized with the SV40 plasmid. Although it is being used as an in vitro model of several respiratory diseases, the potential for the application of the 16HBE cells in nasal permeability studies remains unclear [46]. In culture, and when reaching confluency, 16HBE cells form tight junctional seals, become polar and show apical microvilli, and present the cAMP-regulated CFTR (cystic fibrosis transmembrane conductance regulator, a chloride channel), they are able to develop TEER values similar to the ones seen in the Calu-3 airway epithelial cell line model and normal bronchial epithelia in primary culture [45,46].

## 4. Solid Lipid Nanoparticles (SLN) and Nanostructured Lipid Carriers (NLC) for Nasal Delivery

The inclusion of lipid nanoparticles, such as SLN and NLC, in nasal formulations can improve the effectiveness of drugs. Regarding their advantages over other colloidal carriers, lipid nanoparticles have been described as superior carriers for nasal drug delivery. For instance, they enable the direct transport of drugs from the nose to the brain, via olfactory and trigeminal nerves, and adhere to the olfactory epithelium, increasing contact time with the nasal mucosa. In addition, they provide prolonged drug release, drug protection from nasal enzymatic degradation and have low or no toxicity due to the use of generally recognized as safe (GRAS) excipients [1,2,3,47,48]. 

To understand the specific features of lipid nanoparticles for nasal delivery, it is important to first clarify their specific characteristics. Briefly, SLN were first created and consist of aqueous dispersions of nanoparticles made by one solid lipid and stabilized by one or two emulsifiers. Their solid matrix enables prolonged release, while protecting the encapsulated molecules. Although SLN appear to be effective drug carriers, some drawbacks have been observed, in particular, poor storage stability related to the occurrence of lipid polymorphic transitions that originate molecule release and nanoparticle aggregation. To circumvent these problems, NLCs were developed, which also consist of aqueous dispersions of nanoparticles with a solid lipid matrix composed of one solid lipid and one liquid lipid and stabilized by one or two emulsifiers. The presence of oil within the lipid matrix causes a more disordered internal structure that leads to fewer lipid polymorphic transitions during storage, producing higher stability.

Thereby, the use of SLN and NLC has been extensively investigated to improve drug delivery through different administration routes, as they show advantages over other nanosystems, including the use of GRAS excipients, easy industrial manufacture, high encapsulation efficiency, protection and prolonged release of lipophilic molecules, and good storage stability. In this field, very complete review articles are available [49,50,51,52,53,54,55,56,57,58,59,60,61,62,63].

### 4.1. In Vitro Studies with Nasal Formulations of NLC and SLN

The use of aqueous dispersions of SLN and NLC show limitations in some administration routes, including cutaneous, ocular and nasal. For instance, the low viscosity of these dispersions decreases the contact time with the locale of application, reducing the therapeutic effectiveness of the drug. To avoid this, different strategies have been used, including the incorporation of SLN and NLC in conventional semisolid formulations, such as hydrogels, creams and ointments, or the addition of viscosifying agents, mucoadhesive polymers or in situ gelling polymers, directly to the aqueous phase of the SLN and NLC dispersions [53,64,65,66,67]. Examples of viscosifying agents used in nasal formulations containing lipid nanoparticles include gellan gum, poloxamers, and carbomers [12,68,69], while commonly used mucoadhesive polymers are hypromellose, carbomers, alginate, hyaluronic acid, chitosan, polyethylene glycol, cyclodextrins, polyacrylic acid and cellulose derivatives, such as carboxymethylcellulose, hydroxypropyl methylcellulose and methylcellulose. Examples of in situ gelling polymers include poloxamers, such as poloxamer 407 and 188, gellan gum, pectin, sodium alginate, carrageenan and xyloglucan [12,13,18,20,69,70].

Regarding nasal administration, the use of liquid and semisolid formulations has been investigated and it seems that both formulations promote the efficacy of drugs for different therapeutic applications. In the following sections, examples of the most relevant studies are reported. The main outcomes of these studies are summarized in Table 2. Over the past two years, about ten studies have been published investigating the use of SLN or NLC for intranasal delivery, mainly for the treatment of neurological disorders. 

#### 4.1.1. Liquid Formulations

Du et al. [71] developed ketoconazole-loaded NLC for nose-to-brain delivery in the treatment of cryptococcus neoformans-mediated meningoencephalitis, which is a critical infectious disorder of the CNS. These authors investigated this strategy because the therapeutic effectiveness of conventional treatments is limited due to the poor penetration across the BBB. The developed ketoconazole-loaded NLC presented appropriate particle size, good stability and the fluorescence images demonstrated that the optimized formulations were able to penetrate the C. neoformans capsules. The in vitro antifungal activity against the cryptococcus neoformans was evaluated in the ketoconazole-loaded NLC and ketoconazole solution in fungal cells, using the yeast-extract peptone dextrose and RPMI 1640 medium. The results showed that the fungal growth inhibition was significant at concentrations above 0.5 µg/mL, for the yeast-extract peptone dextrose medium, with a growth inhibition of 92% for ketoconazole-loaded NLC and 50% for ketoconazole solution. In the RPMI 1640 medium, the cell inhibition rate was four-fold higher for the NLC formulation than the ketoconazole solution. Furthermore, the ketoconazole-loaded NLC exhibited greater inhibition rates even at low concentrations, indicating a higher cell uptake.

Jojo et al. [72] evaluated the nasal cytotoxicity of optimized pioglitazone-loaded NLC formulation for the treatment and management of Alzheimer’s disease. This antidiabetic drug has been extensively investigated because the most common cause of dementia in the elderly is a metabolic disorder associated to an impaired brain insulin signalling. SH-SY5Y neuroblastoma cells were used to conduct in vitro studies, evaluating the nasal cytotoxicity of pioglitazone-loaded NLC and pure pioglitazone, through cell viability and the lethal concertation 50 (LC50). Based on the results, the LC50 was 16.626 µg/mL for pure pioglitazone and 17.3874 µg/mL for pioglitazone-loaded NLC. In addition, the cell viability was similar for both formulations, being 69.15% for pioglitazone-loaded NLC and 66.89% for pure pioglitazone at a concentration of 10 µg/mL. The results showed that there was no significant change between the NLC formulation and the pure drug, indicating that pioglitazone-loaded NLC is safe for neuronal cells. In another study, Silva et al. [73] evaluated the in vitro cytotoxicity of tacrine-loaded NLC and tacrine-loaded NLC conjugated to an amphipathic peptide in SH-SY5Y neuroblastoma cell lines. The formulation cytotoxicity was evaluated through the MTT assay and SBR assay. From the results, when comparing the same concentration of empty NLC and tacrine-loaded NLC, the cell viability was similar. However, the cell viability of tacrine-loaded NLC conjugated to an amphipathic peptide at the same concentration decreased dramatically. Therefore, a concentration up to 10 µM of tacrine was considered safe. These results showed that tacrine-loaded NLC is safe for neuronal cells, being a promising formulation for the management of Alzheimer’s disease. Trapani et al. [74] compared the in vitro cytotoxicity of grape seed-derived extract dopamine-loaded SLN, dopamine-loaded SLN and grape seed-derived extract-loaded SLN. The conjugation of dopamine with an antioxidant grape seed-derived proanthocyanidin reduces the oxidative stress observed in Parkinson’s disease. The in vitro studies were carried out in SH-SY5Y neuroblastoma cells and olfactory ensheathing cells. One day after the beginning of the tests, it was observed that none of the formulations presented cytotoxicity to the olfactory ensheathing cells and to the SH-SY5Y neuroblastoma cells, in a concentration range of 18–75 µM and 4–34.5 µM of dopamine and grape seed-derived extract, respectively. Therefore, the authors concluded that the tested formulations can be used to improve Parkinson’s disease therapy.

Wang et al. [75] studied the in vitro efficacy of intranasal *Pueraria* flavone solution, *Pueraria* flavone-loaded SLN, *Pueraria* flavone-loaded SLN modified with borneol and *Pueraria* flavone-loaded SLN modified with borneol and stearic acid. Pueraria flavone is extracted from the *Pueraria thoom sonii* and *Pueraria lobata* and is used for the management of CNS diseases, such as Parkinson’s disease and Alzheimer’s disease. In this study, the cellular uptake of the different formulations was tested in Caco-2 cells. The results showed a higher cellular uptake for *Pueraria* flavone-loaded SLN modified with borneol and stearic acid and *Pueraria* flavone-loaded SLN modified with borneol, when compared to *Pueraria* flavone-loaded SLN and pure *Pueraria* flavone at a concentration of 100 mg/mL, 200 mg/mL and 400 mg/mL of *Pueraria* flavone. In addition, a higher cellular uptake was observed for higher temperatures and higher concentrations. From the results of their study, the authors concluded that the modified SLN containing *Pueraria* flavone could be used to improve the management of neurodegenerative diseases.

Malvajerd et al. [76] developed curcumin-loaded SLN and curcumin-loaded NLC to study their potential for brain delivery in the treatment of CNS disorders. Before performing in vivo experiments, the researchers evaluated the in vitro cytotoxicity of the formulations in mouse fetal fibroblast cells using the MTT assay. The results showed a high cell viability (abound 80%) for curcumin-loaded SLN and for curcumin-loaded NLC at concentrations of 1–10 µg/mL, while a slight decrease in cell viability was observed at higher concentrations (20 µg/mL). Thus, the authors concluded that no remarkable cytotoxicity was observed in any of the tested formulations of lipid nanoparticles containing curcumin.

Khanna et al. [77] evaluated the safety of exposing nalbuphine-loaded SLN to human embryonic kidney cells (HEK-293). In this study, the in vitro cytotoxicity of nalbuphine-loaded SLN was tested in a drug concentration range of 100–1000 µM. The results showed a cell viability of 100% for concentrations of 100, 250 and 500 µM, a viability of 80% for a concentration of 750 µM and a viability of almost 75% for a concentration of 1000 µM. These results suggested that nalbuphine-loaded SLN containing drug concentrations up to 750 µM is safe for use in the management of pain. 

Sarma et al. [78] investigated the in vitro cytotoxicity of two different tenofovir disoproxil fumarate-loaded NLC, one with Tween 80 and the other with Tween 80 and Pluronic F68, in bEnd.3 cells of the cerebral cortex, after 24 h and 72 h of exposure. Similar cell viability was observed for both formulations, and at both times, at concentrations of 5, 10 and 50 µg/mL. After 72 h, cell viability decreased at concentrations of 100 µg/mL for both formulations. From these results, the authors concluded that the use of emulsifiers did not cause differences in cytotoxicity. In addition, at concentrations up to 100 µg/mL, tenofovir disoproxil fumarate-loaded NLCs are safe for intranasal administration. Based on these findings, the use of the tenofovir disoproxil fumarate-loaded NLCs for the treatment of Acquired Immune Deficiency Syndrome (AIDS) was proposed.

#### 4.1.2. Semisolid Formulations

Gadhave et al. [79] developed a carbopol-gellan gum in situ gel containing teriflunomide-loaded NLC for the treatment of gliomas. Gellan gum is a natural anionic polysaccharide capable of forming a hydrogel in the presence of cations in the nasal cavity. In this sense, the objective of using gellan gum, as a gelling agent, and carbopol 974P, as a mucoadhesive polymer, was to increase the contact time of the formulation in the nasal cavity, promoting drug absorption. The antitumor activity of the in situ gel containing teriflunomide-loaded NLC, teriflunomide-loaded NLC and pure teriflunomide was evaluated in human U-87 glioma cells. The results showed that pure teriflunomide and the in situ gel containing teriflunomide-loaded NLC had higher cytotoxicity compared to teriflunomide-loaded NLC. After 48 h, cell viability was 4% for pure teriflunomide, 6% for in situ gel containing teriflunomide-loaded NLC and 48.2% for teriflunomide-loaded NLC, at a concentration of 100 µg/mL. The IC_50_ was 78.5 µg/mL for the teriflunomide-loaded NLC, followed by 7 µg/mL for the in situ gel teriflunomide-loaded NLC and 4.8 µg/mL for the pure teriflunomide. Therefore, it was concluded that the in situ gel containing teriflunomide-loaded NLC and pure teriflunomide were more cytotoxic than teriflunomide-loaded NLC. 

Sun et al. [80] evaluated the in vitro cytotoxicity in RPMI 2650 cells of an in situ gel containing paeonol-loaded SLN, paeonol-loaded SLN, blank SLN and a blank in situ gel, using the MTT method. The cell viability of all tested formulations, in the concentration range of 0.001–10 µg/mL, was higher than 90%, indicating biocompatibility. Additionally, the cell viability of blank SLN and paeonol-loaded SLN, without removing the free emulsifiers used to prepare these formulations, decreased with increasing concentration, with strong cytotoxicity being observed at 1000 µg/mL, presenting cell viability of 24.20% and 25.90%, respectively. Furthermore, the live/dead double staining method showed similar dead cell fluorescence intensity in all tested formulations, which was in agreement with the MTT results and indicated good cell viability.

## 5. Nasal Cavity Models

The deposition of drugs in the nasal cavity upon administration remains challenging. Ensuring drug release to the target area of the nasal cavity is essential to obtain the therapeutic effect. Factors that interfere with the pattern of nasal deposition of drugs include [68,81,82,83]: differences in nasal geometries between individuals, age being fundamental, since adults and children have different lengths and areas of the nasal cavity; nasal application device and the respective flow used; complexity of the structure of the nasal cavity; how the patients administer (e.g., whether or not they are breathing); formulation characteristics (e.g., particle size and viscosity). To overcome these drawbacks, the use of nasal cavity models (or nasal casts) and computational models to predict the deposition of drugs in the nasal cavity have been investigated.

Extensive progress in imaging technology and reconstruction software has enabled the 3D reproduction of the human nasal cavity with the correct geometry and dimensions to visualize drug deposition patterns in specific regions [82,84]. To produce a 3D nasal cast it is necessary to have an image of the human nasal cavity, which can be obtained by computed tomography (CT)-scan or magnetic resonance imaging (MRI) [85,86,87]. 

**Figure 3:**
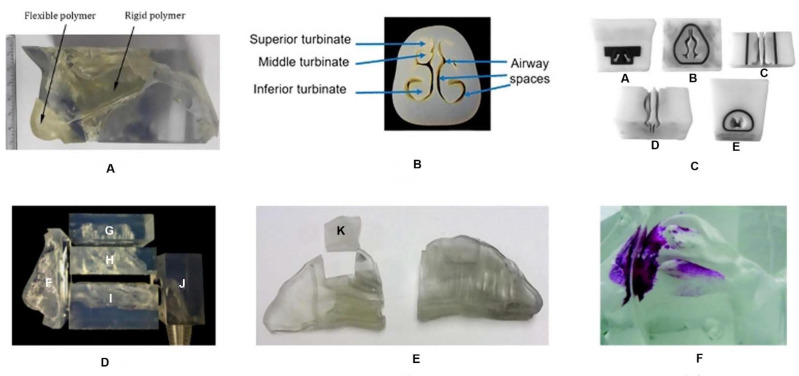
Examples of different nasal casts: (**A**) 7-year-old child; (**B**) MRI image of a 12-year-old child; (**C**) made from CT-scan of an adult; (CA) nostrils, (CB) nasal vestibule, (CC) lower turbinates, (CD) middle and upper turbinates, (CE) nasopharynx; (**D**) CT-scan of a patient, (DF) anterior region, (DG) upper turbinates, (DH) middle turbinates, (DI) lower turbinates, (DJ) nasopharynx; (**E**) MRI image of a 53-year-old man, K—olfactory region; (**F**) silicone transparent commercial cast with Sar-gel (adapted from [90], with permission from Elsevier).

Most 3D models are transparent to allow visualization of the formulation path within the nasal cast (Figure 3). However, it is possible to use a color change method to quantify drug deposition by photometric or colorimetric analysis. Silicone is one of the most used materials to manufacture nasal casts, being described as the most realistic. However, as all nasal casts, silicone casts do not replicate the entire complexity of the nasal cavity, such as nasal valve dynamics or mucociliary clearance. Notwithstanding, the 3D nasal casts allow the visualization of the influence of breathing patterns (with and without airflow), consistency of formulations (liquid, powder or gels), variables of the nasal device (e.g., spray angle and plume characteristics) and the formulation deposition location [68,82,84,87,88,89]. 

**Figure 4:**
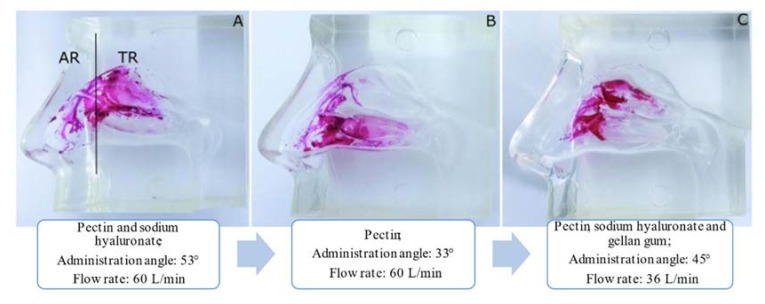
Evaluation of the deposition of fluticasone in situ gels in a 3D nasal cast covered with Sar-gel. (**A**) in situ gels of pectin and sodium hyaluronate, with an administration angle of 53° and a flow rate of 60 L/min; (**B**) in situ gel of pectin, with an administration angle of 33° and a flow rate of 60 L/min, (**C**) in situ gel of pectin, sodium hyaluronate and gellan gum, with an administration angle of 45° and a flow rate of 36 L/min (adapted from [70], with permission from Elsevier).

Nizic et al. [68] used a commercial silicone cast to study the deposition profile of melatonin-loaded pectin/hypromellose microspheres. A respiratory pump was connected to the nasal cast to simulate air inspiration and to observe the differences between inspiratory airflow of 0 L/min and 20 L/min. A nasal insufflator was used to pump the formulation into the nasal cavity cast in one nostril, while the other nostril was closed. Lactose monohydrate was added to increase the fraction of microspheres deposited within the nasal cavity. The results showed a higher drug deposition with an inspiratory flow of 0 L/min than 20 L/min, in all regions of the nasal cavity. It was also observed that the incorporation of lactose monohydrate increased the deposition efficiency in the upper part and in the turbinates of the nasal cavity by 40%, being 8.3 ± 0.2% for the olfactory region and 30.9 ± 4.5% for the turbinates. The same authors [70] evaluated the nasal deposition of in situ gels of fluticasone containing different polymers (sodium hyaluronate, pectin and gellan gum), in the same nasal cast, using different inspiration flow rates (0, 30 and 60 L/min) and different angles of administration (30°, 52.5° and 75°). In addition, Sar-gel, which is an indicator paste that runs purple when it contacts with water, was used to cover the nasal cast and allow visualization of the drug deposition. The results showed that a decrease in the angle of administration from 75° to 30° significantly increased drug deposition in the turbinates, while an increase in the inspiratory flow resulted in drug deposition close to the nasal valve. The use of sodium hyaluronate produced a greater influence on the turbinates deposition pattern, compared to gellan gum. Furthermore, the results of the nasal deposition of in situ gels containing 0.058% of fluticasone and 0.31% of surfactant are shown in Figure 4. Different gelling polymers, angles of administration and inspiratory flow rates were tested. 

From Figure 4, it can be observed that the use of a 3D model of transparent silicone coated with Sar-gel facilitated the visualization of the behavior of the drug in the nasal cast. Furthermore, it was observed that the angle of administration strongly influenced drug deposition between the upper and lower part of the turbinates region, and the combination of three in situ gelling polymers with a low inspiratory flow rate reduced drug deposition in the region of the turbinates.

Recently, the same researchers [85] developed a 3D nasal cast from a CT-scan of a 62-year-old healthy patient, produced by stereolithography using a 3D system and printed on transparent rigid plastic. A respiratory pump was connected to the nasal cast, simulating inspiration, and the differences between an inspiratory airflow of 0 L/min and 20 L/min were analyzed. The Miat spray device was used to administer a dexamethasone sodium phosphate powder formulation to the nasal cavity, with angles of 0°, 60° and 75°. The amount of drug deposited in the olfactory region ranged from 5.1 ± 0.9% to 17.0 ± 1.6%. In addition, it was observed the highest drug deposition with an administration angle of 75° and an inspiratory flow rate of 0 L/min. Gholizadeh et al. [91] compared the drug deposition of a thermosensitive in situ gel of tranexamic acid with a tranexamic acid solution, in the same nasal cast covered with Sar-gel. The results showed that the amount of drug deposited with the thermosensitive in situ gel was 68.52 ± 2.60%, while with the drug solution was 62.79 ± 2.92%. In addition, the deposition pattern remained unchanged from the 20 min for the in situ gel, while for the drug solution it was unstable, showing leakage and runoff. Based on the results, it was concluded that the viscosity of the formulations influenced drug deposition and the use of a mucoadhesive agent increased the residence time in the nasal cavity.

Xi et al. [82] compared the differences in deposition patterns of four commercially available spray pumps (Apotex, Astelin, Miaoling and Nasonex) and four nebulizers (Drive Voyager Pro, Respironics Ultrasonic, Pari Sinus and Philips Respironics) in a nasal cast reconstructed from an MRI scan of an adult male, 3D printed, made of polypropylene and covered with Sar-gel. Higher deposition was observed in the olfactory region with Miaoling, followed by Astelin, Apotex and Nasonex, although most of the deposition occurred in the vestibule and only a small portion reached the upper part of the nasal cavity. Regarding nebulizers, deposition was lower than that of nasal sprays, with Drive Voyager Pro being the one with a higher deposition in the upper part of the nasal cavity. From these results, the authors concluded that the standard nasal delivery systems tested are inadequate to significantly reach the olfactory region of the nasal cavity. In this regard, the same researchers compared the deposition with normal and bidirectional nasal delivery techniques, and the results showed that the latter increased the efficiency of delivery in the olfactory region [92].

Warnken et al. [88] developed ten 3D nasal casts produced from the nasal CT scan of five adults and five children to assess deposition variations related to the nasal geometries and dimensions. The influence of the plume angle (patient-specific angle, 30°, 40°, 60° and 75°) of the nasal sprays on the deposition efficiency in the turbinates was studied. The minimum coronal cross-section areas of the tested nasal casts (corresponding to the nasal valve area in each individual) ranged from 114.0 mm^2^ to 299.2 mm^2^ and the length ranged from 59.2 mm to 88.0 mm. Cromolyn sodium deposition was evaluated in the anterior region, turbinates, upper region and nasopharynx. The results showed a higher deposition of cromolyn sodium in the turbinates, in adults and in children, compared to the deposition in the upper region of the nasal cavity. Furthermore, it was observed that turbinate deposition decreased with increasing the administration angle. In contrast, in the upper region of the nasal cavity, no significant differences were observed with increasing the administration angle, and no deposition was detected in the upper region of some of the nasal casts tested. In addition, a higher deposition of cromolyn sodium in the turbinates was observed in the assessment of patient-specific angle in comparison with the other angles tested, with no significant differences between adults and children. From these results, the authors concluded that nasal sprays are inadequate devices for the efficient administration of drugs in the upper region of the nasal cavity, as the drug was only detected in six of the ten nasal casts tested. Hosseini and Golshahi [93], who also used 3D nasal casts to test drug deposition, obtained different results. These researchers observed the occurrence of higher drug deposition in the olfactory region of adults (3.55 ± 1.29%), followed by children (3.15 ± 0.57%) and toddlers (2.21 ± 0.95%). In addition, higher deposition was also observed in the superior turbinates of adults (2.53 ± 0.88%), followed by children (2.46 ± 0.47%) and toddlers (2.10 ± 0.41%). In this study, researchers also concluded that the use of different nasal delivery devices interferes with drug deposition. They tested two nasal spray pumps (Flonase and Flonase SensimistTM) and one atomization device (MAD nasalTM) and observed the occurrence of olfactory deposition in adults with Flonase and Flonase SensimistTM, being higher with the former. However, the occurrence of olfactory deposition in children and toddlers was not observed with any of the nasal delivery devices tested [94].

### Computational Models

Computational and mathematical approaches to predict the drug deposition in different regions of the nasal cavity are also used to analyze the path followed by nasal formulations upon administration. For instance, Setty [86] developed the eBrain by translating the MRI data into an interactive 3D model that uses graphics and integrates medical images and physiological data. The eBrain allows intranasal drug delivery to be studied under various conditions, predicting the experimental results based on algorithms, design and other set up requirements. In another study, Tian et al. [95] evaluated nasal deposition of inhaled nanoparticles from low to moderate breathing using 3D computer models obtained from the CT scans of a 48-year-old man and a Sprague Dawley rat. This study aimed to visualize the olfactory deposition in nasal cavities with different geometries. For example, in humans the olfactory region comprises about 10% of the nasal cavity, while in rats it occupies about 50% of the nasal cavity. Empirical equations were developed to quantitatively predict the deposition of different nanoparticle sizes under different breathing conditions. 

## 6. Conclusions

The research of novel formulations for nasal drug administration, namely incorporating lipid nanoparticles, such as SLN and NLC, has been attracting the interest of the scientific community. In this respect, the possibility of targeting drugs from the nose to the brain, avoiding the need to cross the BBB, and thus possibly tackling unmet medical needs associated with several CNS disorders, has been a major driver. 

Among the different studies required while engineering novel nasal formulations of lipid nanoparticles, the ones performed with in vitro cell cultures mimicking the nasal epithelium have enabled mechanistic insights into cell uptake, as well as into their cytotoxicity, essential for estimating the safe concentration to be used in the following studies. 

To further test the effectiveness of nasal formulations in reaching the upper part of the nasal cavity, critical for successful nose-to-brain delivery, the use of nasal cavity models encompasses a great potential. Their manufacture, relying on 3D CT scans of the human nasal cavity or computational models of this cavity, has brought about major improvements in the recapitulation of some features of the nasal cavity. They enable analysis of the factors interfering with nasal drug deposition, such as nasal cavity area, type of administration device and angle of application, inspiratory flow rate, presence of mucoadhesive agents, among others. Notwithstanding, they do not preclude the use of confirmatory in vivo studies, a significant impact on the 3R (replacement, reduction and refinement) principle within the scope of animal experiments is expected. The use of 3D nasal casts to test nasal formulations of lipid nanoparticles is still totally unexplored, to the authors’ best knowledge, thus constituting a wide open field of research.

## Figures and Tables

**Table 1:** Characteristics of the different regions of the nasal cavity (data from [3,9,13,15,16,17]).

Region	Surface Area	Location	Characteristics	Vascularization	Epithelium
Vestibule	0.6 cm^2^	Anterior part	Poor permeability and small surface area that limits drug absorption. Presence of mucus and hairs or vibrissae, which constitute an important defense mechanism, preventing the entrance of toxic particles, pathogens and allergens from the external environment into the body.	Low	Squamous epithelium
Respiratory region	130 cm^2^	Middle part and lateral walls	High permeability and large surface area, being the region where the greatest absorption of drugs occurs. Divided into three turbinates: inferior, middle and superior. Provides drug absorption to the systemic circulation. Direct pathway of drug transport to the brain via the trigeminal nerve. Presents cilia, microvilli and mucus. Occurrence of mucociliary clearance mechanism.	High	Respiratory epithelium: ciliated pseudostratified and columnar epithelium
Olfactory region	10 cm^2^	Upper part	Located above the respiratory region and below the cribriform plate. Includes superior turbinate, and a small upper portion of the middle turbinate. Enables drug access from the nose to the brain via the olfactory bulb, bypassing the blood−brain barrier (BBB). Responsible for detecting odors.	High	Olfactory epithelium

**Table 2:** Relevant outcomes from in vitro studies with nasal formulations of solid lipid nanoparticles (SLN) and nanostructured lipid carriers (NLC).

Type of Lipid Nanoparticle Formulation	Drug	Targeted Disease	Cell Line	Relevant Results	Reference
SLN-liquid	Curcumin	CNS disorders	Mouse fetal fibroblasts	High cell viability (80%) for curcumin-loaded NLC and curcumin-loaded SLN, in a concentration range of 1–10 µg/mL. No significant difference in cell viability was observed between the drug-loaded lipid nanoparticles, blank nanoparticles and free curcumin. At a concentration of 20 µg/mL, a slight reduction in cell viability was observed.	[76]
SLN-liquid	Dopamine and grape seed extract	Parkinson’s disease	SH-SY5Y neuroblastoma and Olfactory ensheathing	None of the three formulations (grape seed-derived extract dopamine-loaded SLN, dopamine-loaded SLN and grape seed-derived extract-loaded SLN) presented cytotoxicity to olfactory ensheathing cells and SH-SY5Y neuroblastoma cells, in a concentration range of 18–75 µM and 4–34.5 µM for dopamine and grape seed-derived extract, respectively.	[74]
NLC-liquid	Ketoconazole	Meningoencephalitis	Fungal cells	In the yeast-extract peptone dextrose medium, the fungal growth inhibition effect of ketoconazole-loaded NLC was significant at concentrations above 0.5 µg/mL, having shown a growth inhibition of 92%, compared to a 50% inhibition shown by the ketoconazole solution. In the RPMI 1640 medium, the cell inhibition rate was 4-fold higher for the ketoconazole-loaded NLC formulation than for the ketoconazole solution.	[71]
SLN-liquid	Nalbuphine	Pain management	Human embryonic kidney (HEK-293)	A concentration up to 750 µM was shown to be nontoxic to HEK-293 cells. Percent cell survival was 100% for nalbuphine concentrations of 100, 250 and 500 µM, 80% for a concentration of 750 µM and almost 75% for a concentration of 1000 µM.	[77]
SLN-semisolid	Paeonol	CNS disorders	RPMI 2650	Cell viability of the in situ gel containing paeonol-loaded SLN, paeonol-loaded SLN, blank SLN, and blank in situ gel over a concentration range of 0.001–10 µg/mL was greater than 90%, indicating good biocompatibility. The fluorescence intensity of dead cells was similar for the four formulations tested, indicating good cell viability.	[80]
NLC-liquid	Pioglitazone	Alzheimer’s disease	SH-SY5Y	The LC50 was 16.626 µg/mL for pure pioglitazone and 17.387 µg/mL for NLC loaded with pioglitazone. Cell viability was similar for both formulations, being 69.15% for NLC loaded with pioglitazone and 66.89% for pure pioglitazone at a concentration of 10 µg/mL.	[72]
SLN-liquid	*Pueraria* flavone	CNS disorders	Caco-2	Greater cellular uptake was observed for *Pueraria* flavone-loaded SLN modified with borneol and stearic acid, followed by *Pueraria* flavone-loaded SLN modified with borneol, *Pueraria* flavone-loaded SLN and *Pueraria* flavone free, at 37 °C and 4 °C, at concentrations 100, 200 and 400 mg/mL of *Pueraria* flavone. Cellular uptake of all formulations was achieved at the highest temperatures and concentrations.	[75]
NLC-liquid	Tacrine	Alzheimer’s disease	SH-SY5Y	Blank NLC and tacrine-loaded NLC, at the same concentration, showed similar cell viability. The cell viability of tacrine-loaded NLC conjugated to an amphipathic peptide drastically decreased compared to tacrine-loaded NLC at the same concentration. The use of a concentration up to 10 µM of tacrine was considered safe.	[73]
NLC-liquid	Tenofovir disoproxil fumarate	Acquired Immune Deficiency Syndrome (AIDS)	bEnd.3 cerebral cortex	The two different tenofovir disoproxil fumarate-loaded NLC showed cell viability similar to blank NLC at a concentration of 5, 10 and 50 µg/mL. Cell viability decreased in a concentration of 100 µg/mL of tenofovir disoproxil fumarate after 72 h in both formulations. The use of emulsifiers did not cause any cytotoxicity below 100 µg/mL of tenofovir disoproxil fumarate-loaded NLC.	[78]
NLC-semisolid	Teriflunomide	Glioma	Human U-87	Based on the percentage of viable cells, pure teriflunomide and the in situ gel containing teriflunomide-loaded NLC showed greater cytotoxicity compared to teriflunomide-loaded NLC. After 48 h, cell viability was 4% for pure teriflunomide, 6% for in situ gel, and 48.2% for NLC, for a concentration of 100 µg/mL. The IC50 concentration was 78.5 µg/mL for NLC, followed by the in situ gel at 7 µg/mL and by teriflunomide at 4.8 µg/mL.	[79]

## Data Availability

Data sharing not applicable.
